# Learning receptive awareness via neurofeedback in stressed healthcare providers: a prospective pilot investigation

**DOI:** 10.1186/s13104-018-3756-0

**Published:** 2018-09-04

**Authors:** C. Michael Dunham, Amanda L. Burger, Barbara M. Hileman, Elisha A. Chance

**Affiliations:** 1 Trauma, Critical Care, and General Surgery Services, St. Elizabeth Youngstown Hospital, 1044 Belmont Ave., Youngstown, OH 44501 USA; 2 Behavioral Medicine, St. Elizabeth Family Medicine Residency, 1053 Belmont Ave., Youngstown, OH 44504 USA; 3 Trauma and Neuroscience Research Department, St. Elizabeth Youngstown Hospital, 1044 Belmont Ave., Youngstown, OH 44501 USA

**Keywords:** Bispectral Index, BIS monitor, Neurofeedback, Stress, Mindfulness, Physicians, Nurses

## Abstract

**Objective:**

Because physicians and nurses are commonly stressed, Bispectral Index™ (BIS) neurofeedback, following trainer instructions, was used to learn to lower the electroencephalography-derived BIS value, indicating that a state of receptive awareness (relaxed alertness) had been achieved.

**Results:**

Ten physicians/nurses participated in 21 learning days with 9 undergoing ≤ 3 days. The BIS-nadir for the 21 days was decreased (88.7) compared to baseline (97.0; *p* < 0.01). From 21 wellbeing surveys, moderately-to-extremely rated stress responses were a feeling of irritation 38.1%; nervousness 14.3%; over-reacting 28.6%; tension 66.7%; being overwhelmed 38.1%; being drained 38.1%; and people being too demanding 52.4% (57.1% had ≥ 2 stress indicators). Quite a bit-to-extremely rated positive-affect responses were restful sleep 28.6%; energetic 0%; and alert 47.6% (90.5% had ≥ 2 positive-affect responses rated as slightly-to-moderately). For 1 subject who underwent 4 learning days, mean BIS was lower on day 4 (95.1) than on day 1 (96.8; *p* < 0.01). The wellbeing score increased 23.3% on day 4 (37) compared to day 1 (30). Changes in BIS values provide evidence that brainwave self-regulation can be learned and may manifest with wellbeing. These findings suggest that stress and impairments in positive-affect are common in physicians/nurses.

*Trial Registration* ClinicalTrials.gov NCT03152331. Registered May 15, 2017

**Electronic supplementary material:**

The online version of this article (10.1186/s13104-018-3756-0) contains supplementary material, which is available to authorized users.

## Introduction

For nurses and physicians, concerns exist relative to emotional exhaustion, burnout, and job dissatisfaction. Among medical students, residents/fellows, and early career physicians, adverse manifestation rates were 30–40% for emotional exhaustion, 40–50% for burnout, 40–60% for depression, 7–9% for suicidal ideation, and 50–60% for fatigue [1]. The rate of emotional exhaustion, a risk for burnout, is substantial in nurses in the United States [2, 3]. Surveys have indicated that 20–35% of hospital-based nurses have expressed the intent to leave their current job in the near future [2, 4, 5].

Mindfulness is an attitudinal expression of receptive awareness (relaxed attention), wherein there is a distinction made between a direct experience occurring in the present moment as opposed to associated thoughts and interpretations about the experience [6]. Among physicians and nurses, a high mindfulness score was associated with less stress, greater wellbeing, and a positive emotional tone [7, 8]. Mindfulness training has been associated with reductions in stress or burnout risk in nurses [9–11] and physicians [10–12].

Neurofeedback (NFB) is a process in which an individual learns to intentionally alter their brainwave activity [13]. NFB is useful for decreasing anxiety [14–16] and for enhancing attention [17, 18], mood [19], memory [20], musical performance [21], and surgical technique [22]. Sensors are applied and an electroencephalography (EEG) device computes the power according to frequency bandwidths [13]. A reward bandwidth target is chosen, typically an intermediate range [13, 23]. A computer monitor screen produces visual symbolic images as a mechanism for providing moment-to-moment EEG feedback to the trainee.

Although combining mindfulness and NFB has been advocated [24], such a model has not been evaluated. The purpose of our study was to evaluate a model of mindfulness and NFB to facilitate learning receptive awareness (relaxed attention). As concerns with EEG artifacts and the fact that technologies for providing quantitative EEG analysis are continuously evolving [25], we selected the Bispectral Index™ (BIS) monitor (Aspect Medical Systems, Newton, MA) to provide NFB signals [26, 27]. We hypothesized that receptive awareness or relaxed attention could be learned.

## Main text

Physicians and nurses were welcomed to participate in the learning sessions. Each learning day consisted of two 12-min BIS monitor-NFB sessions. Immediately before session 1, specific instructions were given (Additional file 1). Immediately before session 2, additional detailed instructions were provided (Additional file 2).

Using the manufacturer’s instructions, the BIS sensor was applied to the participant’s left forehead and temporal fossa following a scrubbing of the skin with alcohol and then wiping with a dry cloth. The BIS monitor chart data function was set to 1-min intervals such that the BIS value would be recorded on the monitor hard drive. Awake BIS values were available from the literature and included 685 subjects [28]. The first BIS value (minute 1) on learning day 1 of the first session was also used as a baseline reference for the learners.

A wellbeing surveillance tool was developed after reviewing elements from 5 established systems [29–33]. The physician/nurse learner completed the wellbeing surveillance tool before session 1 on each day. The 7 stress indicators included irritation, nervousness, over-reaction, tension, feeling overwhelmed, feeling emotionally drained, and feeling that people demand too much. The learner rated each indicator based on the prior 3 days as follows: (1) extremely; (2) quite a bit; (3) moderately; (4) a little; and (5) very slightly or not at all. The non-stress score was the sum of each indicator with a range of 7–35.

The 3 positive-affect indicators included restful sleep, feeling energetic, and feeling alert. The learner rated each indicator based on the prior 3 days as follows: (1) very slightly or not at all; (2) a little; (3) moderately; (4) quite a bit; and 5, extremely. The positive-affect score was the sum of each indicator with a range of 3–15. The total wellbeing score was the sum of the non-stress score and the positive-affect score with a range of 10–50, such that a score of 10 suggested extreme stress and 50 suggested little to no stress (Additional file 3).

### Statistical analyses

Data were entered into an Excel 2010 worksheet (Microsoft Corp., Redmond, WA, USA) and imported into the SAS System for Windows, release 9.2 (SAS Institute Inc., Cary, NC, USA). The level of significance was *p* < 0.05. Summary group average values are presented as the mean and standard deviation.

### Results

Ten physician/nurse subjects participated in 21 learning days (May to October 2017). The distribution of subjects according to the number of learning days was as follows: 1 day, 2 subjects; 2 days, 6 subjects, 3 days, 1 subject; and 4 days, 1 subject. The BIS-nadir for the 21 learning days was substantially lower (88.7 ± 3.2) than the awake values described in the literature (96.6 ± 1.7; *p* < 0.0001). The BIS-nadir for the 21 learning days was also lower (88.7 ± 3.2) than the first BIS value on learning day 1 (97.0 ± 0.9; *p* < 0.0001).

Of the 7 stress indicators, moderate-to-extremely responses were as follows: irritation, 8 learners (38.1%); nervousness, 3 learners (14.3%); over-reaction, 6 learners (28.6%); tension, 14 learners (66.7%); overwhelmed, 8 learners (38.1%); drained, 8 learners (38.1%); and people being too demanding, 11 learners (52.4%). Of the 21 learning days, 17 (71.4%) had at least 1 stress indicator and 12 (57.1%) had ≥ 2 stress indicators scored as moderately-to-extremely. Of the 3 positive-affect indicators, quite a bit-to-extremely responses were as follows: restful sleep, 6 learners (28.6%); energetic, 0 learners (0%); and alert, 10 learners (47.6%). Of the 21 learning days, 19 (90.5%) learners had ≥ 2 positive-affects scored as very slightly or not at all-to-moderately. Summary results were as follows: total wellbeing score, 34.4 ± 5.8; non-stress score, 25.4 ± 5.4; and positive-affect score, 9.0 ± 1.6.

One subject underwent 4 learning days over a 19-day period. Mean BIS values were as follows: day 1, 96.8 ± 1.4; day 2, 96.4 ± 1.7; day 3, 95.3 ± 1.8; and day 4, 95.1 ± 2.5. Values were significantly different (*p* < 0.05) for day 1 compared to day 3; day 1 compared to day 4; day 2 compared to day 3; and day 2 compared to day 4. The wellbeing score increased 23.3% on day 4 (37) compared to day 1 (30). The non-stress score increased 30.4% on day 4 (30) compared to day 1 (23).

### Discussion

The lowest BIS value reached during the 21 learning day NFB sessions was substantially decreased, when compared to individuals described in the literature and to the very-first BIS value for each learner. These observations indicate that the learners were effectively able to alter their brainwave activity and enter an attentional state of receptive awareness or relaxed attention by following the instructions.

Data from the 21 wellbeing surveys demonstrated that stress was a common manifestation among the physician-nurse learners. That is, a substantial proportion of learners had perceptions of irritability, over-reaction, tension, feeling overwhelmed, feeling drained, or that people were too demanding. The mean non-stress score for the learners (25.4) represents a 30% reduction relative to the best non-stress score (35). This was an anticipated finding and it suggests that the newly developed surveillance tool may be useful.

Relative to the positive-affect indicators, a large percentage had perceptions that they were deficient in restful sleep, energy, or alertness. The mean positive-affect score for the learners (9.0) is a 40% reduction relative to an ideal positive-affect score (15). Such deficiencies are likely to be manifestations of stress. The mean total wellbeing score, sum of the non-stress and positive-affect scores, for the learners (34.4) represents a 30% reduction relative to a perfect total wellbeing score (50).

Of substantial interest is the 1 learner who underwent 4 learning days. These data indicate that the ability to alter brainwave activity and enhance relaxed alertness is a progressively learned phenomenon. Similarly interesting was the increment in the total wellbeing and non-stress scores on learning day 4 when compared to day 1. These data are consistent with the notion that learning receptive awareness (relaxed attention) might have an influence on daily wellbeing.

#### Attentional focus and stress

Immediately before session 1, the learner was oriented to the BIS monitor screen and before session 2, the learner was told that they should not think too intensely or narrowly focus on the BIS number. Visual perceptual skills are largely influenced by attentional control which consists of executive, orienting, and alerting aspects [34]. Arousal, attention, and stress have an inverted U-shaped relationship with cognitive-motor performance, known as the Yerkes–Dodson law [35, 36]. That is, performance is low with inadequate arousal, attention, and stress but increases with greater arousal, up to a level, where execution efficiencies then decrease with intense focus and excess stress.

Investigators have espoused that narrow attentional visual focus can increase anxiety, muscle tension, autonomic arousal, and hypervigilance [37–39]. Widening the visual scope of attentional focus has been associated with relaxed attention, a balanced state of arousal and sympathetic and parasympathetic neural function [38] and improvements in anxiety [37, 40, 41] and athletic decision making [42]. Others have also provided evidence that enhancing one’s awareness of external space positively increases intermediate brainwave activity [38, 39, 43], relaxation [44], and perceptions of wholeness [45, 46].

#### BIS monitor

The Food and Drug Administration classifies the BIS monitor as a computer device that detects EEG signals and may be used for assessing the clinical and physiological effects of anesthetic and sedating agents. The credibility and validity of the device is supported by more than 2500 citations in the National Library of Medicine that includes publications in the *New England Journal of Medicine* [47] and *Cochrane Systematic Review* [48]. Several studies have demonstrated significant associations between BIS monitor values (0–100) and clinical status, using the Modified Observer’s Assessment of Alertness/Sedation Scale, in patients undergoing general anesthesia [49, 50] or conscious sedation [51]. Reductions in BIS values have also been found for conditions other than pharmacologic sedation and include acupressure [52], stage I sleep [53], and relaxation using guided imagery [54]. For BIS values between 60 and 100, the level highly-correlates (r = 0.90; *p* < 0.01) with the ratio of power in a brainwave band with high frequency (30–47 Hz) relative to an intermediate frequency band (11–20 Hz) [55]. That is, as the BIS value (the symbolic image being displayed) decreases, there is a relative linear reduction in the high frequency brainwave power relative to lower frequency brainwave power.

### Conclusions

The BIS-nadir values provide objective evidence that the self-regulation of brainwave activity can be learned by altering attentional functions. The wellbeing surveys from the physician-nurse suggest that symptoms of stress were relatively frequent and punctuated with impairments in positive-affect. We are planning to provide participant compensation as a mechanism to incentivize learners to participate in at least 4 days of NFB to determine if learning receptive awareness is associated with improvement in wellbeing.

## Limitations

The principal study limitation is that only 1 subject undertook 4 learning days of NFB. Thus, we cannot be certain that the decrease in BIS value and improvement in wellbeing over the 4 learning days will be replicated in a population of learners with similar participation. The wellbeing survey has not been validated using benchmark psychological testing.

## Additional files

**Additional file 1.** Specific instructions given before session 1.

**Additional file 2.** Pre-Session 1 Instructions. Specific instructions given before session 2.

**Additional file 3.** Pre-Session 2 Instructions. Wellbeing surveillance tool and scoring system.

## Abbreviations

BIS: Bispectral Index™
EEG: electroencephalography
NFB: neurofeedback
