# Supplementary material for: Learning receptive awareness via neurofeedback in stressed healthcare providers: a prospective pilot investigation
Source: BMC Res Notes. 2018 Sep 4;11:645. doi: 10.1186/s13104-018-3756-0 (PMC6123908; doi:10.1186/s13104-018-3756-0)
Supplement: Supplementary file 1 — Additional file 1. Specific instructions given before session 1. [file 13104_2018_3756_MOESM1_ESM.doc]

**Additional file 1** Specific instructions given before session 1

Sit comfortably with your hands on your legs.

Keep your eyes open during the session.

View your brainwave activity in the EEG display (trainer pointed to the raw EEG display).

A BIS value more than 94 indicates fast brainwave activity, wherein attention might be associated with stress.

Note that the BIS number might decrease by balancing relaxation with attention.

Be curious to explore the feeling of relaxed attention.
