# Supplementary material for: Learning receptive awareness via neurofeedback in stressed healthcare providers: a prospective pilot investigation
Source: BMC Res Notes. 2018 Sep 4;11:645. doi: 10.1186/s13104-018-3756-0 (PMC6123908; doi:10.1186/s13104-018-3756-0)
Supplement: Supplementary file 2 — Additional file 2. Pre-Session 1 Instructions. Specific instructions given before session 2. [file 13104_2018_3756_MOESM2_ESM.doc]

**Additional file 2** Specific instructions given before session 2

Listen to these suggestions that might foster relaxed attention.

Do not think too intensely.

If thoughts arise, gently return your attention to the activity.

Try not to focus too narrowly on the BIS number and use modest, relaxation.

Simply observe the BIS number, accepting each value as it is.

In addition to looking at the BIS number, be aware of the entire monitor display.

Also, notice the space to the left of the monitor.

Additionally, notice the space to the right of the monitor.

Gently, be aware of the space to the left and right of the monitor.
