# Supplementary material for: Learning receptive awareness via neurofeedback in stressed healthcare providers: a prospective pilot investigation
Source: BMC Res Notes. 2018 Sep 4;11:645. doi: 10.1186/s13104-018-3756-0 (PMC6123908; doi:10.1186/s13104-018-3756-0)
Supplement: Supplementary file 3 — Additional file 3. Pre-Session 2 Instructions. Wellbeing surveillance tool and scoring system. [file 13104_2018_3756_MOESM3_ESM.doc]

**Additional file 3** Wellbeing surveillance tool and scoring system

| **For the past 3 days, rate your level of the following:** | **Very slightly or not at all** | **A little** | **Moderately** | **Quite a bit** | **Extremely** |
| --- | --- | --- | --- | --- | --- |
| **Restful sleep** |  |  |  |  |  |
| **Feeling energetic** |  |  |  |  |  |
| **Feeling alert** |  |  |  |  |  |
| **Irritation** |  |  |  |  |  |
| **Nervousness** |  |  |  |  |  |
| **Over-reaction** |  |  |  |  |  |
| **Tension** |  |  |  |  |  |
| **Feeling overwhelmed** |  |  |  |  |  |
| **Feeling emotionally drained** |  |  |  |  |  |
| **Feeling that people demand too much** |  |  |  |  |  |

**Wellbeing Scoring System**

| **Positive Affects:** |  | **Stress Indicators:** | |
| --- | --- | --- | --- |
| **Restful Sleep**  **Energetic**  **Alert** |  | **Irritation**  **Nervousness**  **Over-reaction**  **Tension** | **Overwhelmed**  **Drained**  **Demanding people** |
| - 1=very slightly or not at all - 2=a little - 3=moderately - 4=quite a bit - 5=extremely |  | - 1=extremely - 2=quite a bit - 3=moderately - 4=a little - 5=very slightly or not at all | |
